# Metabolic Syndrome and Triple-Negative Breast Cancer: A New Paradigm

**DOI:** 10.1155/2012/809291

**Published:** 2011-10-15

**Authors:** Andrew A. Davis, Virginia G. Kaklamani

**Affiliations:** Cancer Genetics Program, Division of Hematology/Oncology, Department of Medicine and Robert H. Lurie Comprehensive Cancer Center, Feinberg School of Medicine, Northwestern University, Chicago, IL 60611, USA

## Abstract

Triple-negative breast cancers (TNBCs) are aggressive tumors with poor prognosis compared to other breast cancer subtypes. The evidence linking TNBC with the metabolic syndrome, which consists of central obesity, insulin resistance, impaired glucose tolerance, dyslipidemia, and hypertension, has emerged from clinical studies and experiments using cell lines and mouse models. Epidemiological studies have associated abdominal obesity with increased incidence of TNBC. Additionally, insulin resistance, dyslipidemia, and hypertension are associated with increased incidence of breast cancer across all subtypes. The insulin-leptin-adiponectin axis has been implicated mechanistically in breast cancer tumorigenesis. Specifically, increased leptin and decreased adiponectin levels disrupt homeostatic signaling pathways involved in cell proliferation, survival, cell-cycle regulation, and angiogenesis. Insulin, insulin-like growth factor I (IGF-I), and epidermal growth factor receptor (EGFR) may mediate interactions between these two hormones. Further research will facilitate the development of targeted therapeutics and programs to modify lifestyle factors to modulate the insulin-leptin-adiponectin axis for TNBC.

## 1. Introduction

Triple-negative breast cancers (TNBCs) lack expression of the steroid receptors estrogen (ER) and progesterone (PR) and the tyrosine kinase human epidermal growth factor receptor 2 (HER-2). Therefore, TNBCs are a diagnosis of exclusion, typically characterized by upregulation of cytokeratins 5, 14, and 17 and elevation of the epidermal growth factor receptor (EGFR) [1–3]. Studies estimate that approximately 15–20% of breast cancers meet these criteria [4–6]. Compared to other breast cancer subtypes, TNBCs are typically aggressive, invasive (ductal, medullary, or metaplastic), grade III tumors with high rates of mitotic division, of which approximately half contain a high rate of p53 mutations [7]. For these reasons, they account for a disproportionately high percentage of metastases, distant recurrence, and death among patients with breast cancer. Metastases in TNBCs are most common to visceral organs including liver, lungs, and central nervous system. As a diagnosis of exclusion, TNBC overlaps considerably with basal-like breast cancer (BLBC) although differences between the two subtypes exist, especially at a genetic level. Other molecular subtypes defined by gene expression patterns include luminal A, luminal B, HER-2-enriched group, and claudin-low, all of which may include TNBCs to some extent [8, 9]. TNCBs are most common among premenopausal women, especially those of African American descent [4–6, 10]. In addition, TNBCs are common among patients with BRCA1 mutations [11, 12]. 

Since the first molecular characterization of TNBCs in the literature in 2005, the topic has quickly emerged as an active area of research [13]. While initial studies focused on molecular and clinical characterizations of patients with the diagnosis, more recent studies have identified subgroups of patients with TNBC, proposed molecular mechanisms that may contribute to tumorigenesis, and explored potential therapeutic interventions for patients. In this paper, we examine the connection between TNBC and the metabolic syndrome, which consists of central obesity, insulin resistance, impaired glucose tolerance, dyslipidemia, and hypertension. Our analysis of the literature will encompass *in vitro* and *in vivo* studies in cell lines and mouse models of TNBC, respectively, as well as clinical studies examining epidemiology and treatment of TNBC.

## 2. Risk Factors for TNBC 

Obesity, which is associated with insulin resistance and type 2 diabetes mellitus (DM), is an established risk factor for cancer incidence. In a meta-analysis of 141 articles, body mass index (BMI) was positively associated with an increased incidence of postmenopausal breast cancer, along with colon, endometrial, esophageal, gallbladder, pancreas, renal, thyroid cancers, leukemia, multiple myeloma, and non-Hodgkin's lymphoma in women [14]. The results were less clear, however, for premenopausal breast cancer as a positive association between obesity and premenopausal breast cancer was found in Asia-Pacific women (risk ratio (RR) = 1.16; 95% CI, 1.01–1.32), while inverse relations were reported in North American women (RR = 0.91; 95% CI, 0.85–0.98) and European and Australian women (RR = 0.89; 95% CI, 0.84–0.94) These findings suggest that different subpopulations of women possess different risk factors for breast cancer. It may also suggest that BMI is not an ideal measure of adiposity. Instead, other measures such as waist-to-hip ratio (WHR) or waist circumference, which are specific measures of central or abdominal adiposity, may be preferential to assess cancer risk. Two meta-analyses that examined a correlation between elevated WHR and risk of breast cancer in premenopausal women reported positive associations [15, 16]. The study by Connolly et al. reported that elevated WHR was associated with a 79% (summary risk (SR) = 1.79; 95% CI, 1.22–2.62) increased risk of breast cancer for premenopausal women and a 50% (SR = 1.50; 95% CI, 1.10–2.04) increased risk for postmenopausal women [15]. Similarly, the study by Harvie et al. reported that small WHR was associated with a 37% decreased risk (RR = 0.63, 95% CI, 0.45–0.88) in premenopausal women only after adjusting for BMI [16]. The authors hypothesized that general obesity may not modulate risk, but central obesity increases risk in premenopausal women. In contrast, the authors reported that general obesity and not central obesity increased cancer risk in postmenopausal women. This interesting result led the authors to hypothesize that insulin resistance and insulin-like growth factors, which are associated with central obesity, may play a larger role in modifying breast cancer risk for premenopausal women, while estrogen may play a greater role in postmenopausal breast cancer [16]. 

While the link connecting obesity and incidence of all types of breast cancers is well established, the data examining obesity and TNBC are much less prevalent. In the Carolina Breast Cancer Study, WHR was compared between the highest (≥0.84) and lowest (<0.77) groups in relation to BLBC [17]. Across all women, there was an increased risk (odds ratio (OR) = 2.3; 95% CI, 1.4–3.6) for developing BLBC with higher WHR. Premenopausal women (OR = 1.8; 95% CI, 1.0–3.4) and postmenopausal women (OR = 2.7; 95% CI, 1.3–5.4) with high WHR both had elevated risk of developing breast cancer compared to the lowest WHR group. Weight gain in women as reported since fifth grade was highest in African American women in this sample. In contrast, no significant trend was reported for BMI and risk of breast cancer. A 2008 study examining 620 predominantly white women in rural Appalachia, 117 of whom had TNBC, reported a significant association between obesity and incidence of TNBC [18]. In this sample, approximately 50% of patients with TNBCs were obese as compared to 36% of non-TNBCs. Obesity in this study was defined as a BMI ≥30. The preponderance of evidence suggests an association between TNBC and obesity when obesity is defined as an elevated WHR, but more contradictory evidence exists when using BMI as a measure of obesity. Clearly, the conflicting results warrant additional research. Future epidemiological studies would benefit from measurement of all three receptor markers and studies that concurrently examine multiple definitions of obesity.

A common corollary of metabolic syndrome, type 2 DM, has been associated with increased risk of breast cancer. A 2007 meta-analysis of twenty studies estimated a 20% increased risk of breast cancer for women with type 2 DM (RR = 1.20; 95% CI, 1.12–1.28) [19]. For TNBC, one study reported a significant relation with 58% of patients with TNBC possessing a comorbid diagnosis of metabolic syndrome compared to 37% of patients without TNBC in a sample of 176 individuals using criteria of the National Cholesterol Education Program and 52% compared to 34% using criteria of the American Association of Clinical Endocrinologists [20]. In addition, a 2011 study reported a 75% increase in the risk of postmenopausal breast cancer (RR = 1.75; 95% CI, 1.37–2.22) for women who were found to have at least three of the four components of metabolic syndrome [21]. However, the Carolina Breast Cancer Study reported no elevated prevalence of type 2 DM in TNBC compared to other breast cancer subtypes [17].

Recently, epidemiological studies have associated dyslipidemia and hypertension with breast cancer risk. In a prospective study examining all-cancer incidence of 1,189,719 Korean men and women, Kitahara et al. reported a positive association between total cholesterol and breast cancer risk in women (hazard ratio (HR) = 1.17; 95% CI, 1.03–1.33) [22]. The researchers compared individuals with total cholesterol ≥240 mg/dL to individuals with cholesterol  <160 mg/dL and adjusted for cigarette smoking, alcohol consumption, BMI, fasting serum glucose, hypertension, and physical activity. In addition, hypertension was independently predictive of breast cancer risk in a sample of 3,869 postmenopausal women with breast cancer as compared to 4,082 controls (OR = 1.19; 95% CI, 1.07–1.33) [21]. Another study reported a 23% increased risk of breast cancer for hypertensive women [23]. However, after adjustment of confounders including BMI, the elevated risk was no longer significant (HR = 1.14; 95% CI, 0.93–1.40). 

Epidemiological studies suggest a positive association between the metabolic syndrome as a whole, along with many of its individual components, and breast cancer risk. The many confounding variables that may mediate this effect need to be considered in order to determine whether this is a causative effect. Studies would benefit from multi-institution designs to assess geographically diverse populations. Further studies should also address how changes in components of metabolic syndrome, such as weight, affect incidence of disease, and treatment outcomes after initial diagnosis of TNBC. Larger sample sizes will determine whether subpopulations of patients with TNBC (e.g., pre- versus postmenopausal women) possess unique clinical and molecular characteristics.

## 3. Risk of Recurrence and Mortality in TNBC

In addition to exploring risk factors that influence incidence of TNBC (primary prevention), it is also essential to understand factors that influence recurrence of TNBC (secondary prevention). Compared to other subtypes of breast cancer, TNBCs are more often diagnosed as aggressive, invasive, grade III, and lymph-node positive tumors [7]. These outcomes are predictive of increased morbidity and mortality. In addition, TNBCs have a high rate of recurrence with visceral metastases compared to other subtypes of breast cancer, especially within the first five years after diagnosis [24]. After five years, the risk of recurrence drops dramatically. 

Obese patients with breast cancer have more frequent recurrence and worse prognosis as compared to lean patients. In a sample of 495,477 U.S. women, increasing BMI was significantly associated with increased death rates for breast cancer [25]. As compared to the lowest BMI group (18.5–24.9), there was an elevated risk of 34% for BMI of 25.0–29.9 (RR = 1.34; 95% CI, 1.23–1.46), 63% for BMI of 30.0–34.9 (RR = 1.63; 95% CI, 1.44–1.85), 70% for BMI of 35.0–39.9 (RR = 1.70; 95% CI, 1.33–2.17), and 112% for BMI ≥ 40.0 (RR = 2.12; 95% CI, 1.41–3.19) of dying of breast cancer. Furthermore, in a sample of 18,967 patients in Denmark with early-stage breast cancer, BMI at diagnosis was correlated with disease prognosis. Patients with BMI ≥ 30 kg/m^2^ had a 46% higher risk of distant metastases (HR = 1.46; 95% CI, 1.11–1.92) after 10 years and 38% increased risk of mortality from breast cancer (HR = 1.38; 95% CI, 1.11–1.71) ) as compared to patients with BMI < 25 kg/m^2^ [26]. The authors also suggested that adjuvant chemotherapy and endocrine therapy were less effective over time periods greater than 10 years for patients with BMI > 30 although it was unclear whether this effect was mediated by poor responsiveness to treatment or differences in biology. Even though obese patients were more likely to present with advanced tumors in terms of size and spread to lymph nodes, obesity was still an independent predictor after controlling for these confounders. A recent, single institution study examined BMI in 418 patients treated for TNBC [27]. The study measured BMI after diagnosis of TNBC and then counted the number of recurrences and deaths. After controlling for clinically significant factors, no significant relation was found between BMI and overall survival (HR = 0.94; 95% CI, 0.54–1.64) or recurrence-free survival (HR = 0.81; 95% CI, 0.49–1.34). In a sample of 1,169 patients diagnosed with invasive breast cancer, the relationship between general obesity and response to neoadjuvant chemotherapy was examined [28]. When comparing overweight (BMI 25 to <30 kg/m^2^) and obese (BMI ≥ 30 kg/m^2^) groups to the normal/underweight group (BMI < 25), a significant association was present for pathologic complete response to neoadjuvant chemotherapy (OR = 0.67; 95% CI, 0.45–0.99) in the normal/underweight group. While high BMI was associated with worse overall survival, no significant effects were seen for breast-cancer specific or progression-free survival. Finally, although data linking risk of recurrence and mortality in patients with hypertension and TNBC are limited, a 2011 study retrospectively examined the use of beta blockers on prognosis for patients with breast cancer [29]. After adjustment for a number of covariates, patients with TNBC who were taking beta blockers had significantly improved relapse-free survival (HR = 0.30; 95% CI, 0.10–0.87), and while overall survival was improved (HR = 0.35; 95% CI, 0.12–1.00), it only approached a significance level (*P* = 0.05). Similar findings were also reported for non-TNBC subtypes.

A number of epidemiological studies have suggested that physical activity and weight loss are inversely related breast cancer risk and recurrence. The Women's Healthy Eating and Living (WHEL) Study prospectively examined 1,490 women with breast cancer [30]. The authors reported that performing exercise equivalent to walking 30 min, six days per week, and consuming ≥5 daily servings of fruits and vegetables decreased mortality by 46% (HR = 0.56; 95% CI, 0.31–0.98). While ER+ tumors were associated with decreased mortality with these lifestyle interventions (*P* < 0.05), no significant effect was observed for ER−, PR− tumors (*P* = 0.40). To the best of our knowledge, the largest study to date examining the link between physical activity and invasive breast cancer was a meta-analysis of 12,108 patients, which included six studies [31]. While physical activity prior to diagnosis had no effect on breast cancer deaths across all patients, physical activity after diagnosis reduced breast cancer deaths by 34% (HR = 0.66, 95% CI, 0.57–0.77) and disease recurrence by 24% (HR = 0.76, 95% CI, 0.66–0.87). Postdiagnosis exercise only provided significant benefits for patients with BMI ≥ 25 kg/m^2^. Interestingly, physical activity after diagnosis reduced breast cancer deaths by 50% (HR = 0.50, 95% CI, 0.34–0.74) for ER+ tumors with no significant effect for patients with ER− tumors. When looking at the individual studies that composed the meta-analysis, the studies that examined postdiagnosis physical activity were prospective, observational, and questionnaire-based studies, while those that examined prediagnosis physical activity had case-control designs [32–37]. While the definition of physical activity varied somewhat from study to study, the studies generally defined physical activity as moderate recreational activity, and for the purpose of their analyses, the authors combined these forms of exercise into metabolic equivalent task (MET) hours per week. Examples of moderate physical activity included walking, jogging, running, biking, swimming, tennis, calisthenics/aerobics, and squash/racquetball. One study included in the meta-analysis specifically examined the relation between risk reduction of breast cancer and duration of exercise [32]. In a sample of 2,987 women diagnosed with breast cancer, the number of hours an individual exercised per week was categorized. Compared to women who performed the equivalent of walking at an average pace less than 3 MET-hours per week, there was a nonsignificant 20% risk reduction of death from breast cancer for 3 to 8.9 MET-hours per week (RR = 0.80; 95% CI, 0.60–1.06), a significant 50% risk reduction for 9 to 14.9 MET-hours per week (RR = 0.50; 95% CI, 0.31–0.82), 44% risk reduction for 15 to 23.9 MET-hours per week (RR = 0.56; 95% CI, 0.38–0.84), and 40% risk reduction for 24 or more MET-hours per week (RR = 0.60; 95% CI, 0.40–0.89). This study, however, did not find a significant effect for exercise, even for 9 or more MET-hours per week, for ER−, PR− tumors. 

These studies provide an insight on the role of physical activity as a potentially beneficial breast cancer treatment that may be used in conjunction with existing radiation and chemotherapy treatments [32–37]. Although studies explicitly targeting patients with TNBC have not been performed, a potential mechanism behind this link may be decreased concentrations of estrogen via reduction in body fat or decreased androgens via increase in globulins that bind testosterone [38]. Improvements in insulin resistance or blood glucose may also mediate this effect. 

In addition to exercise, two large randomized studies have examined whether diet interventions are effective in reducing breast cancer recurrence and mortality [39, 40]. The Women's Intervention Nutrition Study (WINS) examined 2,437 women with breast cancer [39]. The randomized study involved a dietary intervention group with a goal of reducing calories from fat to 15% without compromising nutrition compared to control with median followup of 60 months. The intervention group had statistically lower fat intake (*P* < 0.001). When comparing relapse events between the two groups, relapse was lower in the intervention group as compared to the control group (HR = 0.76; 95% CI, 0.60–0.98, *P* = 0.077 for stratified log rank and *P* = 0.034 for adjusted Cox model analysis). The authors reported a trend for a stronger effect for dietary fat reduction for hormone receptor-negative cancers (HR = 0.58; 95% CI, 0.37–0.91) compared to ER+ tumors (HR = 0.85; 95% CI, 0.63–1.14), although no significant effect was found (interaction test, *P* = 0.15). One of the criticisms of the WINS study was the fact that the intervention group lost about 6 pounds more than the control arm over the duration of the study (*P* = 0.005). As a result, it was unclear whether the outcomes were due to decreased weight or decreased fat intake. Furthermore, the dietary intervention was relatively strict, making it hard to implement in everyday practice. In addition, the WHEL study evaluated the potential benefit of physical activity and a diet rich in vegetables and fruit in breast cancer survivors [40]. The study included 3,088 women with early-stage breast cancer. The arm randomized to a diet rich in vegetables, fruit, and fiber, but low in fat did not have a significantly lower mortality (HR = 0.91; 95% CI, 0.72–1.15) or a lower incidence of second invasive breast cancer (HR = 0.96; 95% CI, 0.80–1.14) during a 7.3-year follow-up period. In this study, the intervention and comparison groups had an average weight difference of 1-kg or less based on measurements at baseline, 1 year, 2 or 3 years, 4 years, and 6 years. In an analysis of the comparison group only, consuming ≥5 daily servings of fruits and vegetables and performing exercise equivalent to walking 30 min, six days per week at baseline was associated with lower mortality [30]. No effect, however, was reported in the randomized trial based on physical activity at baseline for additional breast cancer events or all-cause mortality. These conflicting findings warrant further research, especially to assess diet interventions for patients with TNBC. 

Alcohol consumption also appears to moderate recurrence and mortality for breast cancer survivors. In a recent study of 1,897 individuals, consumption of three to four alcoholic drinks or more per week was associated with a 35% (HR = 1.35; 95% CI, 1.00–1.83) increased risk of breast cancer recurrence and 51% (HR = 1.51; 95% CI, 1.00–2.29) increased risk of death due to breast cancer [41]. No difference was found between ER+ versus ER− subgroups although the authors noted that this lack of effect may have been due to a small sample size of patients with ER− tumors. Further studies will be important to assess whether different subtypes of breast cancer are affected differently by diet and alcohol in order to further probe the mechanism of these effects.

## 4. Insulin and TNBC

Insulin is implicated as a link between obesity and breast cancer risk. In particular, upregulation of insulin has been hypothesized to directly increase proliferation of breast tissue and breast cancer cells. A 2009 study, which measured insulin at baseline and at 1, 3, and 6 years of followup, reported a HR of 2.22 (95% CI, 1.39–3.53) for incidence of breast cancer in postmenopausal women when comparing the highest baseline insulin concentration group to the lowest group [42]. Another study demonstrated that a high homeostatic model assessment score, which is associated with serum levels of insulin and glucose, was correlated with increased breast cancer mortality in a sample of 527 women [43]. Samples were collected at a single time point, 30 months postdiagnosis. Similarly, a 2011 study of 604 women in the Health, Eating, Activity, and Lifestyle (HEAL) Study measured serum C-peptide, a marker of insulin secretion, three years after diagnosis [44]. An increased C-peptide concentration of 1 ng/mL was associated with a 35% increased risk of death from breast cancer (HR = 1.35; 95% CI, 1.02–1.87). Collectively, these data suggest that hyperglycemia and hyperinsulinemia are associated with poor prognosis for patients with breast cancer. In contrast, a 2007 case-control study examining blood samples in predominantly premenopausal women reported that increased levels of insulin and C-peptide were not risk factors for breast cancer [45]. This study, however, did not examine ER−, PR− tumors. A recent study by Erickson et al. examined type 2 DM and associated prognosis in patients with breast cancer [46]. Baseline hemoglobin A1C (HbA1C) levels among 3,003 patients were examined for recurrence and all-cause mortality. The authors reported a significant increase in all-cause mortality after adjustment for confounders for women with HbA1C ≥7.0% as compared to <6.5% (HR = 2.35; 95% CI, 1.56–3.54). 

The actions of insulin may also occur indirectly via decreased availability of globulin and insulin-like growth factor- (IGF-) binding proteins and increased blood concentration of testosterone, estrogens, or IGFs. Elevated concentrations of unbound estradiol and testosterone have been associated with increased breast cancer risk in pre- and postmenopausal women [47–50]. These compounds have been proposed as molecular links between obesity and breast cancer risk. Insulin also inhibits sex hormone-binding globulin (SHBG) production and increases the levels of IGF-I in blood, which results in increased mitogenic activity [51]. This link is consistent with approximately 50% of breast cancer tumors overexpressing IGF-I receptor [52]. A recent laboratory study found that seven cell lines that serve as models of TNBC expressed IGF receptors [53]. Surprisingly, expression was at similar levels to ER+ cell lines even though type I IGF receptor levels are increased by estrogen in ER+ cell lines. In all cases, IGF-I increased proliferation and survival of the cancer cell lines. 

Although studies have reported a positive association between type 2 DM and breast cancer, a potential confounding variable in establishing this relation is treatment regimen [54]. Insulin has recently been implicated to have cancer promoting effects, while recent evidence suggests metformin to have cancer protecting effects in patients with type 2 DM [55]. Most patients with type 2 DM are prescribed either insulin or metformin. Insulin glargine use, especially when prolonged, may increase the incidence of breast cancer. In one study, this effect was especially prominent for individuals who had received insulin for an average of 5.6 years before starting insulin glargine (HR = 2.7; 95% CI, 1.1–6.5) [56]. In contrast, metformin has been shown to inhibit proliferation and colony formation of TNBC cells *in vitro *[57]. Further experiments extended these findings into *in vivo *mice. Metformin resulted in decreased tumor growth if injected in TNBC tumor xenograft mice and decreased tumor incidence if added before injecting TNBC cells. While the molecular mechanism of how metformin reduces breast cancer incidence and survival is unclear, potential mechanisms include (1) acting as a general growth inhibitor, (2) reducing serum insulin levels, and (3) reducing body weight [54, 57]. Interestingly, the drug only exhibited an antiapoptotic effect in TNBC cell lines, an effect which was not present for luminal A, B, and HER-2 subtypes [58]. Recently, observational studies were performed suggesting that metformin reduces the risk of breast cancer in humans. In one study, metformin use was associated with a 38% lower incidence of ER+, PR+ tumors in postmenopausal women with type 2 DM [59]. No significant effect was demonstrated for ER−, PR− tumors, however, although the sample size for TNBCs was limited. In addition, prospective studies are under way on the role of metformin in breast cancer recurrence. Further studies are necessary to determine whether elevated levels of insulin and C-peptide are risk factors for women with TNBC, as well as to elucidate the mechanism behind this association. 

## 5. Leptin and TNBC

Leptin is the product of the obesity (ob) gene and is primarily synthesized and secreted by adipose tissue, with increasing adiposity associated with higher circulating leptin levels. [60]. Leptin helps regulate food intake and metabolism via its actions on the arcuate nucleus of the hypothalamus. It is hypothesized that leptin resistance in obese individuals may be analogous to insulin resistance in diabetics [61]. This resistance has been proposed to develop via impaired transport of leptin across the blood brain barrier and circumventricular organs and leptin receptor signal attenuation [62]. Clinical studies have reported a positive association between circulating blood leptin and breast cancer risk with particular elevation of mRNA expression in adipocytes in close proximity to the tumor [63]. 

On a molecular level, it has been hypothesized that elevated leptin expression in epithelial mammary cells may promote tumorigenesis via mechanisms including cell proliferation (aromatase, MAPK, STAT3, and cyclin D1), angiogenesis (VEGF), apoptosis (p53 and caspase 9), cell-cycle regulation (p21), and cell survival (Akt) in breast cancer cell lines [64]. In TNBC cell lines, a study by Saxena et al. reported that leptin directly increased activity of the IGF-I receptor [65]. Similarly, IGF-I reciprocally increased activity of the leptin receptor via phosphorylation. In addition, bidirectional crosstalk between leptin and IGF-I upregulated EGFR promoting proliferation and migration of TNBC cells. The study further reported that using the EGFR inhibitors, lapatinib and erlotinib, in an *in vitro *model system for metastasis after application of leptin and IGF-I reduced invasion and migration of breast cancer cells [65]. Collectively, these data suggest a possible therapeutic route for treatment of TNBC with EGFR inhibitors, because up to 70% of TNBCs overexpress EGFR [7]. In addition to leptin and IGF-I, a 2011 study by Burga et al. reported another potential mechanism for elevated levels of EGFR protein [66]. After RNA knockdown of BRCA1 in mammary epithelial cells, EGFR protein was upregulated due to transcriptional modification and posttranslational stabilization of EGFR. This is important to our understanding of TNBCs, because BRCA1 mutations are highly correlated with TNBCs. Interestingly, EGFR inhibition with erlotinib in female BRCA1 knockout mice, *in vivo*, prevented or delayed development of ER−, but not ER+ tumors. However, the treatment was not effective in shrinking the tumor after tumorigenesis [66]. 

A causal link between leptin and breast cancer is supported by animal studies in which obese mice that overexpressed transforming growth factor-alpha (TGF-*α*), but were deficient in leptin, did not develop mammary tumors, while heterozygous and homozygous wild type leptin mice developed tumors in 50% and 67% of cases, respectively [67]. However, these findings were difficult to interpret, because leptin deficient mice possessed limited mammary tissue. Further studies in mouse models, *in vivo*, suggest a therapeutic potential for leptin receptor, antagonists. In a recent study of 69 TNBC tumors, 92% of breast tumors expressed leptin receptor and 86% expressed leptin [68]. In this study, the peptide Allo-aca, a leptin receptor antagonist, extended survival time by up to 80% in a TNBC mouse xenograft model,* in vivo*. Clinical studies are needed to determine whether leptin antagonists may hold promise as a therapy in humans, especially in obese patients who overexpress leptin.

Clinical trials in humans are currently underway to test the efficacy of EGFR inhibitors in TNBC. These studies have focused on using cetuximab, a humanized antiEGFR IgG1 antibody in conjunction with ixabepilone, cisplatin, carboplatin, or a taxane. (NCT00633464, NCT00463788, [69–71]). In one study, 12 patients with metastatic TNBC were treated with either paclitaxel or docetaxel with cetuximab weekly [69]. Of the eleven patients assessable to followup, nine (82%) exhibited decrease in size of metastasis, but three (27%) developed brain metastasis during treatment (133). Other studies by Carey et al. and O'Shaughnessy et al. have reported therapeutic value of using EGFR inhibitors in conjunction with other chemotherapy agents including (1) carboplatin plus cetuximab and (2) irinotecan and carboplatin, plus cetuximab [70, 71]. The study by Carey et al. compared cetuximab alone to carboplatin plus cetuximab in patients with TNBC metastases [70]. Of the 71 patients who received both drugs, 13 (18%) responded to treatment as compared to only 2 of 31 (6%) of patients who received cetuximab alone. In addition, the preliminary results of the randomized phase II study of metastatic patients with TNBC by O'Shaughnessy et al. reported no improvement in objective response rate (ORR), progression-free survival, and overall survival across all patients with metastatic disease when comparing cetuximab in conjunction with irinotecan and carboplatin as compared to irinotecan and carboplatin [71]. However, subset analysis of revealed that ORR was increased in metastatic patients with TNBC when using all three drugs (19 of 39; 49%) as compared to only irinotecan and carboplatin (10 of 33; 30%). These findings may suggest a therapeutic benefit of using EGFR inhibitors for a subset of patients with TNBC. Larger experimental and control groups and increased number of follow-up years will benefit our understanding of the potential for these treatments.

## 6. Adiponectin and TNBC

Adiponectin, a protein secreted exclusively by adipose tissue, is an endogenous insulin sensitizer. Levels of adiponectin are inversely correlated with obesity. In contrast to the procarcinogenic effects of leptin, adiponectin may possess anticarcinogenic effects. After controlling for BMI, studies have reported that women with increased adiponectin concentrations possessed a 65% reduced risk for breast cancer [72–74]. In another sample of 527 women diagnosed with stage I–IIIA breast cancer, adiponectin levels above 15.5 *μ*g/mL were associated with improved breast cancer survival (HR = 0.39; 95% CI, 0.15–0.95) [43]. Interestingly, in a 2011 study by Oh et al. the authors reported prognostic value of adipokines in ER−, PR− tumors but not ER+, PR+ tumors (*P* for trend =0.027) [75]. Patients with low adiponectin levels as defined by the first quartile in the study had a significantly increased likelihood of cancer recurrence as compared to patients in the fourth quartile (HR = 2.82; 95% CI, 1.03–7.68). These results were significant even after adjustment for BMI and homeostasis model assessment scores for insulin resistance. Serum leptin levels were not correlated with diseased outcome in this study. Genetic data also links adiponectin to breast cancer risk. We recently evaluated the role of adiponectin pathway single nucleotide polymorphisms (SNPs) in breast cancer risk. We performed a case-control study on 733 breast cancer cases and 839 controls and genotyped 10 haplotype-tagging SNPs of adiponectin (*ADIPOQ*) and the type I adiponectin receptor (*ADIPOR1*) genes [76]. We showed that two functional polymorphisms of *ADIPOQ*, and one functional polymorphism which has been shown to alter mRNA levels of *ADIPOR1* was significantly associated with risk of breast cancer. When categorized by signaling status, low adiponectin signalers had a 6.56-fold increase in breast cancer risk (95% CI, 0.78–54.89), and intermediate adiponectin signalers had a 4.16-fold increase in risk (95% CI, 0.49–35.19) compared to high signalers (*P* for trend =0.001). Although these data are preliminary, they provide evidence for a significant role for adiponectin in predicting breast cancer risk. 

The mechanisms underlying the association between adiponectin and breast cancer risk have been studied by several investigators. Components of the adiponectin signaling pathway have been implicated in breast tumorigenesis. More specifically, a number of compounds related to cell proliferation (aromatase, MAPK, and cyclin D1), apoptosis (Bcl2 and caspase 8), cell-cycle regulation (AMPK), and cell survival (Akt) have been implicated to mediate tumorigenesis in breast cancer cell lines [64]. While adiponectin has been shown to have an antiproliferative effect on cell growth in both ER+ and ER− cell lines, the dominant mechanisms responsible for these effects in ER+ and ER− cell lines are likely different [72]. For example, in MCF-7 cells, 24 hour treatment with adiponectin resulted in an antiproliferative effect lasting up to 96 hours [77]. Whether adiponectin induces cell apoptosis is controversial and depends on the particular breast cancer cell line and the duration of the adiponectin incubation period [64]. One study reported that increased cleavage of poly (ADP-ribose) polymerase (PARP), which serves as an early apoptotic biomarker, was only detected in ER+ cell lines [78]. Other studies have reported that adiponectin inhibits aromatase and estrogen receptor activity, mechanisms which would primarily act on ER+ tumors [64]. Collectively, these data suggest that adiponectin acts via multiple signaling pathways with different mechanisms predominating in ER+ and ER− cell lines.

Animal studies have demonstrated that overexpression of adiponectin, both locally and systemically, reduces mammary tumor size [79]. In contrast, reduced expression of adiponectin accelerates tumor onset and progression [80]. The proposed mechanisms linking low adiponectin levels and breast carcinogenesis are (1) interaction with insulin [60, 81], (2) interaction with leptin [64], (3) inhibition of TNF-*α* in macrophages [82], (4) binding of fibroblast growth factor and platelet-derived growth factor-beta polypeptide [82], (5) inhibition of nuclear factor *κ*B [83], and (6) promotion of angiogenesis [84]. Further research exploring the link between adiponectin levels over time and breast cancer risk is needed in order to elucidate dominant mechanisms in different breast cancer subtypes. Furthermore, monitoring changes in adiponectin levels in conjunction with different pharmacological and/or behavioral modifications such as diet or exercise in human patients may contribute to a better understanding of its role in TNBC. Finally, treatments aimed at increasing adiponectin levels should be explored for their potential therapeutic and preventive benefit in breast cancer.

## 7. Conclusions

Considerable evidence links the components of metabolic syndrome, including central obesity, insulin resistance, glucose intolerance, dyslipidemia, and hypertension, with the different breast cancer subtypes. Although data on the connection between TNBC and the metabolic syndrome are limited, several studies have provided evidence for this association. Studies have reported an association between elevated abdominal obesity, as defined by a high WHR, and increased incidence of TNBC, but the evidence for BMI is more contradictory [17, 18]. In addition, while type 2 DM and insulin resistance are associated with elevated breast cancer incidence, early evidence suggests that TNBCs do not have increased prevalence of type 2 DM compared to non-TNBCs [17]. In terms of disease progression, obesity is associated with worse prognosis and increased recurrence across all breast cancer subtypes [25, 26, 28]. Hyperglycemia and hyperinsulinemia have also recently been associated with increased incidence and poor prognosis [42–44]. Additionally, behavioral modifications including moderate physical activity, a diet rich in fruits, vegetables, and micronutrients, and reduced alcohol consumption show promise across all breast cancer subtypes [32–37, 39, 41]. It remains to be seen whether these alternative therapies may prove useful in conjunction with chemotherapy for patients with TNBC. 

Molecular mechanisms of how these components of metabolic syndrome may mediate tumorigenesis and disease progression have been proposed. Insulin may mediate breast cancer risk via both direct and indirect effects, resulting in increased concentration of androgens and estrogens, along with increased concentration of IGF-I [47–53]. Leptin and adiponectin, which are both secreted by adipose tissue and often by breast tumors, act via a number of downstream signaling pathways involved in cell proliferation, apoptosis, cell-cycle regulation, angiogenesis, and cell survival [64]. It is likely that normal cells must maintain a fine balance between leptin and adiponectin in order to maintain proper cell and tissue homeostasis, and the components of metabolic syndrome appear to disrupt this balance by increasing leptin and decreasing adiponectin levels [61, 62, 64]. In addition, insulin, IGF-I, and EGFR may play a pivotal role in mediating the potential interactions between these two hormones [65, 66]. 

**Figure 1:**
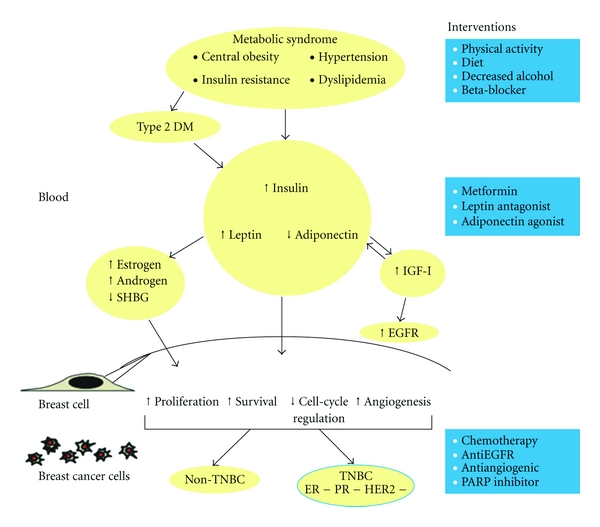
The insulin-leptin-adiponectin axis and risk of TNBC. Schematic representation demonstrates interactions of components in blood. After the compounds enter a normal breast cell, changes in proliferation, survival, cell-cycle regulation, and angiogenesis result in tumorigenesis of either TNBC or non-TNBC. Potential interventions for TNBC, at different levels, are included on the right.

We propose that components of the metabolic syndrome and the insulin-leptin-adiponectin axis play a pivotal role in the pathogenesis and progression of TNBC (Figure 1). At present, treatments for TNBC are limited compared to other subtypes of breast cancer, because these tumors are resistant to hormone therapy and drugs that target the HER-2 protein. Clinical trials have shown efficacy of treatments such as chemotherapy, anti-EGFR drugs, antiangiogenic drugs, and PARP inhibitors in the treatment of TNBC [7]. Lifestyle factors including diet, reduced alcohol consumption, and physical activity, which may modulate components of the metabolic syndrome, may also play a pivotal role in decreasing incidence and risk of recurrence of TNBC. Trials that incorporate agents such as metformin or leptin antagonists as well as other therapies that modify the insulin-leptin-adiponectin axis may prove very beneficial for prevention and treatment of TNBC.
